# Security Analysis of Image Encryption Based on Gyrator Transform by Searching the Rotation Angle with Improved PSO Algorithm

**DOI:** 10.3390/s150819199

**Published:** 2015-08-05

**Authors:** Jun Sang, Jun Zhao, Zhili Xiang, Bin Cai, Hong Xiang

**Affiliations:** 1 Key Laboratory of Dependable Service Computing in Cyber Physical Society of Ministry of Education, Chongqing University, Chongqing 400044, China; E-Mails: taizixzl@gmail.com (Z.X.); caibin@cqu.edu.cn (B.C.); xianghong@cqu.edu.cn (H.X.); 2 School of Software Engineering, Chongqing University, Chongqing 401331, China; 3 School of Automation, Huazhong University of Science and Technology, Wuhan 430074, China; E-Mail: zhaojunhust@gmail.com

**Keywords:** gyrator transform, image encryption, rotation angle, intelligent search, particle swarm optimization (PSO)

## Abstract

Gyrator transform has been widely used for image encryption recently. For gyrator transform-based image encryption, the rotation angle used in the gyrator transform is one of the secret keys. In this paper, by analyzing the properties of the gyrator transform, an improved particle swarm optimization (PSO) algorithm was proposed to search the rotation angle in a single gyrator transform. Since the gyrator transform is continuous, it is time-consuming to exhaustedly search the rotation angle, even considering the data precision in a computer. Therefore, a computational intelligence-based search may be an alternative choice. Considering the properties of severe local convergence and obvious global fluctuations of the gyrator transform, an improved PSO algorithm was proposed to be suitable for such situations. The experimental results demonstrated that the proposed improved PSO algorithm can significantly improve the efficiency of searching the rotation angle in a single gyrator transform. Since gyrator transform is the foundation of image encryption in gyrator transform domains, the research on the method of searching the rotation angle in a single gyrator transform is useful for further study on the security of such image encryption algorithms.

## 1. Introduction

Optical image encryption is one of the main research fields for image encryption. The basic idea is to apply random operations in optical transform domains to encrypt images. One of the typical optical image encryption techniques is the double-random phase-encoding (DRPE) technique [1]. By employing independent random phase encoding on both the input and the Fourier planes, it encodes an input image to a complex-amplitude encoded image, whose real and imaginary parts can be taken as independent stationary white noise. Following the basic idea, the image can also be encrypted in fractional Fourier transform domains [2,3,4,5]. In addition, Chen *et al*. proposed an optical image encryption method by using multilevel Arnold transform and noninterferometric imaging [6]. Reference [7] proposed ghost imaging using labyrinth-like phase modulation patterns for optical image encryption, which is highly efficient and highly secure, since only one phase-only mask should be pre-set and the labyrinth patterns occupy only a few spaces, while each labyrinth pattern possesses high randomness and flexibility. In [8], by employing a nonlinear optical correlation via a sparse phase, phase encryption with enhanced security was proposed.

Gyrator transform is a new optical transform which has been widely studied in recent years [9]. By employing the unique characteristics of gyrator transform, gyrator transform has been used for image encryption, including single image encryption [10,11,12,13,14], double image encryption [15,16,17,18,19,20], multi-image encryption [21], and color image encryption [22,23,24,25,26,27,28].

Image encryption in gyrator transform domains usually applies random operations in image gyrator transform domains to encrypt the secret image [10,11,12,13,14,15,16,17,18,19,20,21,22,23,24,25,26,27,28]. For example, random phase encoding may be applied in image gyrator transform domains [10,11,12]. In such a cryptosystem, since the rotation angle involved in gyrator transform is also one of the secret keys for image encryption, the security is higher than that of the traditional DRPE technique in the Fourier transform domain [1].

To analyze the security of image encryption in gyrator transform domains, the security of image encryption with single random phase encoding and a single gyrator transform was analyzed in [29]. For such a cryptosystem, the operation of random phase encoding only changes the phase angle of the original secret image instead of the amplitude of the image. Thus, the encryption security depends on the rotation angle used in a gyrator transform. Based on the periodicity of gyrator transform, [29] indicated that, by equally dividing the period interval [0, 2π] of a gyrator transform with enough precision, and applying an exhaustive search, the rotation angle used in a gyrator transform can be obtained approximately with a known-plaintext attack. Then, the secret image can be decrypted with various qualities in terms of the dividing precision of the interval [0, 2π]. The analysis results may be used as a starting point for further security analysis of image encryption in gyrator transform domains.

Reference [29] focused on exhaustively searching the rotation angle used in a single gyrator transform. However, since a gyrator transform is continuous, it is time-consuming to exhaustively search for the rotation angle, even considering the periodicity of a gyrator transform and the data precision in a computer. Thus, the computational intelligence method may be applied to speed up the searching.

In this paper, based on [29], by analyzing the properties of gyrator transform, an improved particle swarm optimization (PSO) algorithm was proposed to search for the rotation angle in a single gyrator transform. The research on the properties of a gyrator transform and the method of searching the rotation angle in a single gyrator transform is useful for further study on the security of the image encryption in gyrator transform domains.

This paper is organized as follows: in Section 2, gyrator transform is briefly introduced and analyzed. Section 3 proposes the improved PSO algorithm to search the rotation angle in single gyrator transform, while Section 4 performs the numerical simulation experiment. The final conclusions are presented in Section 5.

## 2. Brief Introduction and Analysis on Gyrator Transform

### 2.1. Gyrator Transform

Gyrator transform is a linear canonical integral transform, which introduces the rotation in position−spatial frequency planes of phase space. For a two-dimensional function $f_{i}({\vec{r}}_{i})$, the gyrator transform with rotation angle α is as Equations (1) and [9]:

(1) $$\begin{array}{l} f_{0}({\vec{r}}_{0})=R^{\text{α}}[f_{i}({\vec{r}}_{i})]({\vec{r}}_{0})=\iint f_{i}(x_{i},y_{i})K_{\alpha }(x_{i},y_{i},x_{0},y_{0})dx_{i}dy_{i}\\ =\frac{1}{\left|\sin\text{α}\right|}\iint f_{i}(x_{i},y_{i})\left\{\exp\left[i2\text{π}\frac{\left(x_{0}y_{0}+x_{i}y_{i})\cos\text{α}-(x_{i}y_{0}+x_{0}y_{i}\right)}{\sin\text{α}}\right]\right\}dx_{i}dy_{i} \end{array}$$

where ${\vec{r}}_{i}$ and ${\vec{r}}_{0}$ are the input and output plane coordinates, respectively. α is the rotation angle. 

For α = 0 it corresponds to the identity transform, for α = π/2, it reduces to the Fourier transform with a rotation of the coordinates at π/2. For α = π, the reverse transform described by the kernel δ (*r*_o_ + *r*_i_) is obtained. Meanwhile for α = 3π/2, it corresponds to the inverse Fourier transform with a rotation of the coordinates at π/2 [9].

The gyrator transform is periodic with 2π. The gyrator transform can be implemented with numerical methods, according to the following three steps to transform $f_{i}(x,y)$ to $f_{o}(x,y)$:

Step 1:

(2) Bα(xi,yi)=fi(xi,yi)exp(j2πxyiicotα)

Step 2:

(3) $$t=\left\{ \begin{array}{l} FFT2(B_{\text{α}}(x_{i},y_{i}))\;if\;\sin\text{α}\geq 0\\ IFFT2(B_{\text{α}}(x_{i},y_{i}))\;if\;\sin\text{α}<0 \end{array}\right.$$

where FFT and IFFT are fast Fourier transform and inverse Fourier transform, respectively.

Step 3:

(4) fo(xi,yi)=texp(j2πxyiicotα)

### 2.2 Analysis on Gyrator Transform

Suppose an image f(x,y) is transformed to g(x,y) with a gyrator transform. The rotation angle used in the gyrator transform is α. With known f(x,y) and g(x,y), we want to find the value of α. 

Due to the continuity of the gyrator transform, the number of the potential values of α used in the gyrator transform is infinite. However, as [29] indicates, since the gyrator transform is periodical with the period of 2π, the value of α may be obtained approximately by discretizing the interval [0, 2π] with enough precision and applying an exhaustive search.

**Figure 1:**
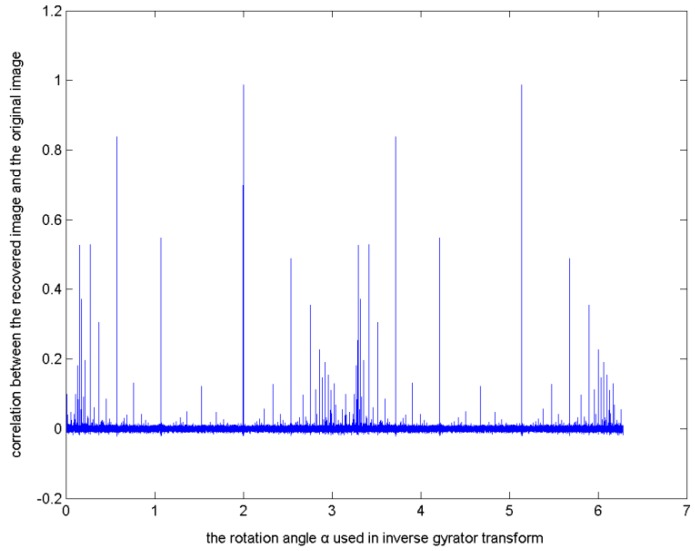
Correlations between the recovered images and the original image by equally dividing [0, 2π] into 100,000 sub-intervals.

For example, apply a gyrator transform on an image, using 2 as the value of the rotation angle. Then, equally divide the interval [0, 2π] into 100,000 sub-intervals and use the obtained discrete values as the rotation angle in an inverse gyrator transform to recover the image. The correlations between the recovered images and the original image were shown in Figure 1 [29].

In Figure 1, the horizontal axis is the value of the rotation angle used in the inverse gyrator transform, while the vertical axis is the correlation between the recovered image and the original image.

From Figure 1, it can be seen that, if the interval [0, 2π] is equally divided into sub-intervals with great enough numbers, *i.e.*, discretized with enough precision, e.g., 100,000, with an exhaustive search, the rotation angle used in a gyrator transform can be obtained approximately and the original image can be recovered with high quality (the correlation being close to 1), even though the obtained rotation angle may not be the same as the original one.

In [29], it is also indicated that, the obtained approximate rotation angle can be used to recover other images transformed with the same rotation angle. It demonstrated that, taking a single gyrator transform as a cryptosystem, by discretizing the interval [0, 2π] with enough precision and applying an exhaustive search, the rotation angle can be obtained with a known-plaintext attack.

Obviously, due to the continuity of the gyrator transform, to obtain the rotation angle used in a single gyrator transform, the interval [0, 2π] should be discretized with enough precision to apply an exhaustive search. It is time-consuming, even considering the data precision in a computer. To accelerate the search speed, by analyzing the properties of the gyrator transform, we proposed an improved PSO algorithm to search the rotation angle used in a single gyrator transform, which will be described in the next section.

## 3. Improved PSO Algorithm

Particle Swarm Optimization (PSO) is a kind of evolution algorithm. It sets a swarm of particles, where each particle represents a potential solution. By evaluating each particle with the value of the fitness function, the velocity and the position of each particle are updated. The procedure of fitness-value evaluation and particle updating are repeated iteratively until an acceptable solution has been found or the maximum number of iterations is reached [30].

Since the gyrator transform has severe local convergence and obvious global fluctuations (Figure 1), it is easy to fall into a locally-optimal solution by applying the typical PSO algorithm to search for the rotation angle in a single gyrator transform. To solve such problem, an improved PSO algorithm to be suitable for such situation was proposed.

The search scope of the proposed improved PSO algorithm is dynamically changed, which is similar to using a microscope. Firstly, the rough position of the solution is observed in a large scope, usually, in the global scope. Then, by adjusting the lens, the solution is observed in a smaller scope, *i.e*., in a local scope. This procedure may be repeated to gradually reduce the search scope until the final solution is either found or not. If the solution is not found after a certain number of iterations, amplify the observing scope for the next loop to try to search the solution again. 

In the improved PSO algorithm, the basic idea of the PSO algorithm is kept, while the search stages corresponding to different search scopes are determined by the current best-fitness value.

Next, the specific procedure for searching the rotation angle in a single gyrator transform based on the improved PSO algorithm is described.

The definitions of the symbols used in the algorithm are listed in Table 1.

**Table 1:** The symbols used in the algorithm.

Symbol	Definition
NLoop	The maximum number of loop
NCLoop	The number of current loop
NGlobal	The maximum iterative number for global search
NCGlobal	The number of current global search
NLocal	The maximum iterative number for local search
NCLocal	The number of current local search
NParticle	The number of particle
Fitout	The fitness value when an acceptable solution is found
SerX	The sizes of local scope for local search X maybe 1, 2, 3, ..., n, which is corresponding to the different sizes of local scope, since the local searching scope maybe getting smaller during the search procedure
FitX	The threshold values of the fitness corresponding to different local search scopes SerX
Fitbest	Current best fitness value
Xbest	Current best particle corresponding to Fitbest

**Figure 2:**
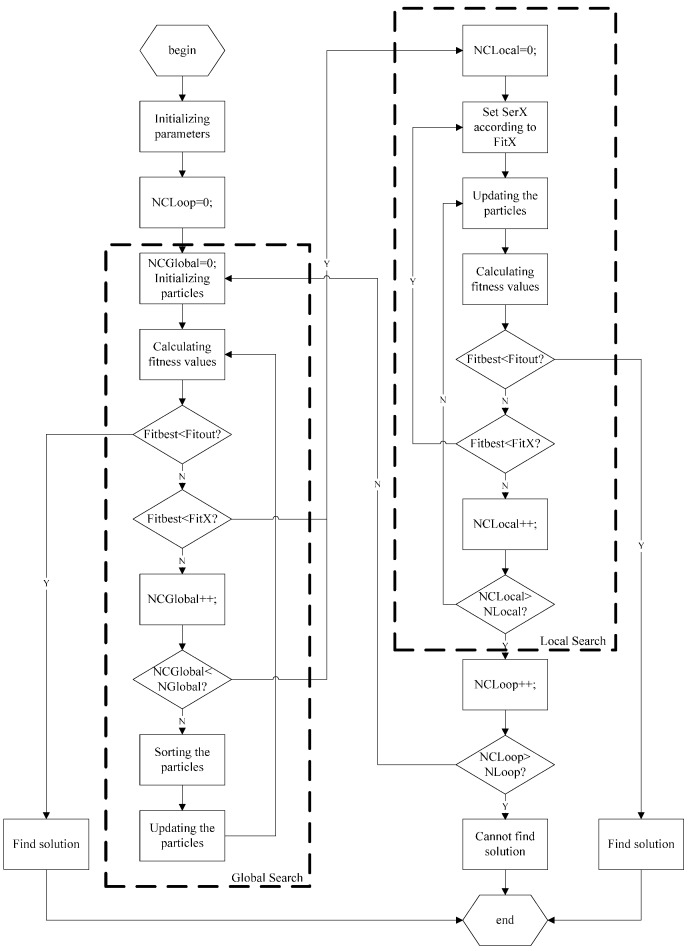
The flowchart of searching the rotation angle in a single gyrator transform with the improved PSO algorithm.

The flowchart of the algorithm is shown in Figure 2.

As Figure 2 illustrates, the procedure of the algorithm can be described as four major stages:

Stage 1: Initialize the parameters used in the algorithm, such as NLoop, NGlobal, NLocal, NParticle, Fitout, SerX, FitX, *etc*. Set NCLoop to be 0.

Stage 2: Global search.

In this stage, globally search the solution, *i.e.*, the rotation angle used in a single gyrator transform. 

Firstly, the particles are initialized by being randomly generated among the interval [0, 2π].

Secondly, the fitness values of the particles are calculated. The fitness function is the mean-square-error (MSE) between the recovered image $f^{\prime }(x,y)$, by applying an inverse gyrator transform on the transformed image, g(x,y) with the particle and the original image f(x,y), which is defined as Equation (5):

(5) $$MSE=\frac{1}{MN}\sum_{x=0}^{M-1}\sum_{y=0}^{N-1}{\left|g(x,y)-f(x,y)\right|}^{2}$$

If Fitbest < Fitout, the solution is found, output it and exit. 

If Fitbest < FitX, go to local search with search scope SerX, and the current best particle Xbest is set to be the particle X(1) used for local search. 

If NCGlobal > NGlobal, go to local search with search scope Ser1, and the current best particle Xbest is set to be the particle X(1) used for local search. This means that if the maximum iterative number for global search NGlobal is reached, even if the threshold value SerX for any local search is not reached, the algorithm will also go to local search.

Then, sort the particles according to their fitness values from least, and the particles are updated following Equation (6).

(6) $$x_{i+1}(j)=\left\{ \begin{array}{l} x_{i}(j)\;1\leq j\leq 5\\ x_{i}(1)+0.001\times \text{rand}(1)\;6\leq j\leq 10\\ x_{i}(2)+0.01\times \text{rand}(1)\;11\leq j\leq 15\\ x_{i}(3)+0.1\times \text{rand}(1)\;16\leq j\leq 20\\ 2\times \text{π}\times \text{rand}(1)\;21\leq j\leq NParticle \end{array}\right.$$

where $x_{i}(j)$ is the position of current particle, $x_{i+1}(j)$ is the position of the particle after updating, and NParticle is the number of particle. 

As Equation (6) shows, the updating strategy for the global search can be described as keeping the five best particles, randomly generating five particles around each of the three best particles, and randomly generating the other particles globally.

Stage 3. Local search. 

In this stage, locally search the solution in a reduced scope.

The local search scope is determined by SerX corresponding to FitX as the interval [X(1) − SerX, X(1) + SerX]. 

Firstly, the particles are updated following Equation (7).

(7) $$x_{i+1}(j)=\left\{ \begin{array}{l} x_{i}(1)\;j=1\\ x_{i}(1)+\text{SerX}\times \text{rand}(1)\;2\leq j\leq 5\\ x_{i}(1)-\text{SerX}+(2\times \text{SerX})/(NParticle-5)\times (j-6)\;6\leq j\leq NParticle \end{array}\right.$$

where $x_{i}(j)$ is the position of current particle, $x_{i+1}(j)$ is the position of the particle after updating, NParticle is the number of particle. 

As Equation (7) shows, the updating strategy for local search can be described as keeping the best particle, randomly generating four particles around the best particle, uniformly generating the other particles in the interval [*x_i_*(1)-SerX, *x_i_*(1)+SerX]. 

Then, calculate the fitness values of the particles.

If Fitbest < Fitout, the solution is found, output it and exit. 

If Fitbest < FitX, where the FitX is less than the threshold value used in the current local search, locally search again in a smaller scope, and the current best particle Xbest is set to be the particle X(1) used for next search. Therefore, during the local search stage, the search scope may be getting smaller. 

If NCLocal <= NLocal, repeat local search with current search scope. 

If NCLocal > NLocal, the solution is not found during the local search. 

Stage 4. Loop again or exit. 

If NCLoop <= NLoop, go to global search to repeat search again. 

If NCLoop > NLoop, cannot find the solution and exit. 

The experiments by applying the proposed improved PSO algorithm to search the rotation angle in a single gyrator transform were conducted in the next section. 

## 4. Experiments and Analysis

The experiments were conduct under the following environment: 

Computer: HP G42 Notebook

CPU: Intel Core i3 M390 @2.67 GHz

OS: Windows 8 Professional

Matlab: R2013a

The values of the parameters used in the experiments are listed in Table 2. 

**Table 2:** The values of the parameters used in the experiments.

Parameter	Value
NLoop	20
NGlobal	40
NLocal	10
NParticle	100
Fitout	0.05
SerX	0.001, 0.0001, 0.00001, 0.000001, 0.0000005
FitX	100, 80, 60, 40, 20

**Figure 3:**
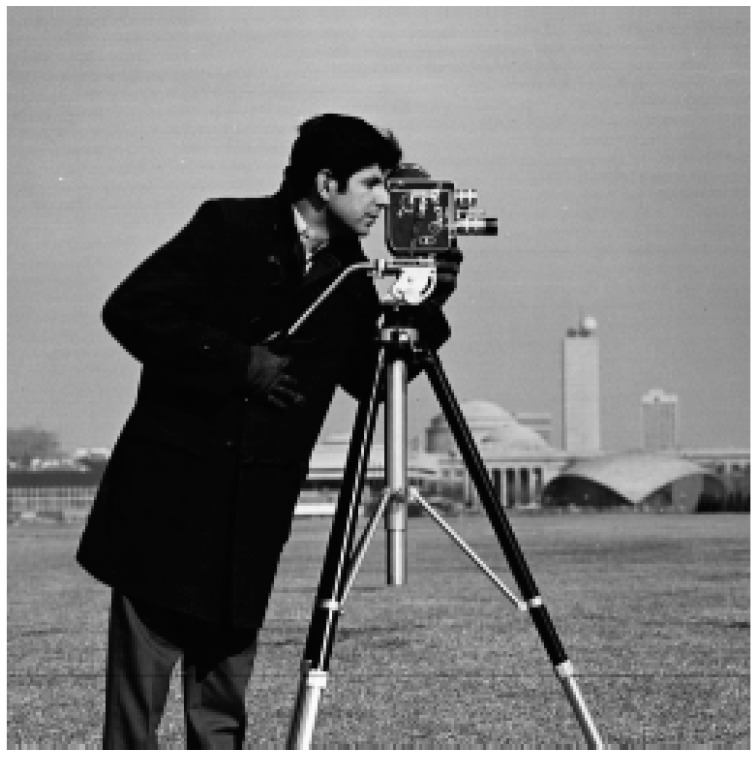
Cameraman.

The image used in the experiments was image Cameraman shown in Figure 3. Different values of the rotation angle in the gyrator transform were used to conduct the experiments several times. The experimental results were listed in Table 3.

**Table 3:** The experimental results for image Cameraman with different values of rotation angle.

NGlobal	Original Rotation Angle	Obtained Rotation Angle	Fitness Value (MSE)	Number of Iteration	Used Time (s)
40	1.723456	0.868545572	0.00016785	63	156.425792
		2.427506487	0.0094	78	194.970638
		1.723456005	0	217	490.312829
		0.496425161	0.0090	77	198.694923
		2.905347971	0.0294	117	298.661654
		2.70692431	0.0285	117	291.795749
	2.523456	1.956698353	0	74	197.288178
		2.523456	0	38	98.478521
		1.035001755	0	78	198.249360

**Figure 4:**
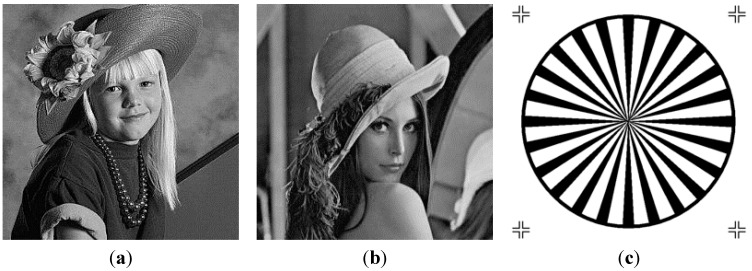
(**a**) Image Girl; (**b**) Image Lena; (**c**) Image Testpat.

In order to compare, image Cameraman and the other three images Girl, Lena, and Testpat, shown in Figure 4, were used in the experiments and the results were list in Table 4.

**Table 4:** The experimental results for four images.

Image	Original Rotation Angle	Obtained Rotation Angle	Fitness Value (MSE)	Number of Iteration	Used Time (s)
Camerama	1.123456	0.594282984	0.000015259	38	103.03
Girl	1.123456	1.12345599	0	88	240.76
Lena	1.123456	2.050489581	0	50	119.18859
Testpat	1.123456	1.123456002	0	65	164.377708

In Table 3 and Table 4, the number of iterations may be larger than NGlobal plus NLocal; because for each loop, the number of iterations is NGlobal plus NLocal. If the solution is not found, the algorithm will loop again. If the solution is found with several loops, the number of iterations is added together. On the other hand, when the maximum number of loops is reached and the solution is not found yet, it means that the solution cannot be found. According to a large number of experiments, by setting the maximum number of loops to 20, the ratio of not obtaining the solution is around 10%–15%.

To compare the time used for searching the rotation angle in a single gyrator transform, the experimental results for the above four images by using the improved PSO algorithm and the exhaustive search were listed in Table 5. Notice that for the improved PSO algorithm, the used time depends on the parameters and the original rotation angle. Here, we listed the used time for general situations following Table 3 and Table 4. For the exhaustive search, according to [29], to obtain the rotation angle in a single gyrator transform, the interval [0, 2π] should be divided into enough sub-intervals, e.g., the value of the rotation angle is accurate to six decimal places. Therefore, the time used for searching the rotation angle in a single gyrator transform was in terms of setting the value of the rotation angle to be accurate to six decimal places.

**Table 5:** The time used for searching the rotation angle in a single gyrator transform.

Image	Improved PSO Algorithm (s)	Exhaustive Searching (s)
Camerama	less than 500	around 71,200
Girl	less than 500	around 71,200
Lena	less than 500	around 71,200
Testpat	less than 500	around 71,200

From Table 3, Table 4 and Table 5, it can be seen that the time to obtain the rotation angle in a gyrator transform with the improved PSO algorithm is usually less than 500 s. On the contrary, if the value of the rotation angle is accurate to six decimal places, with an exhaustive search, the search time will be around 71,200 s. Obviously, with the improved PSO algorithm, the time used to search the rotation angle in a single gyrator transform decreases significantly.

More experiments were conducted on many different images with different rotation angles and different parameters, and similar results were yielded. In addition, our experiments demonstrated that, for different images transformed by a single gyrator transform with the same rotation angle, the rotation angle obtained with an image via the improved PSO algorithm can also be used to recover the other images. It indicates that, taking a single gyrator transform as a cryptosystem, the rotation angle which plays the role of secret key, can be efficiently obtained with a known-plaintext attack via the improved PSO algorithm.

It should be pointed out that the parameters used in the algorithm, such as NLoop, NGlobal, NLocal, NParticle, Fitout, SerX and FitX, *etc*. may be adjusted to be different values and the results may be different. However, in general, the search time with the proposed improved PSO algorithm is usually much less than that of exhaustive search.

From the above experimental results and discussion, it can be seen that encrypting an image with a single gyrator transform is insecure. Therefore, the image encryption methods in the gyrator transform domain usually apply iterative gyrator transforms [10,11,12,13,14,15,16,17,18,19,20,21,22,23,24,25,26,27,28]. With iterative gyrator transforms, the encryption security may be enhanced. However, following the existing security analysis methods for double-random-phase-encoding-based image encryption [31,32,33,34,35,36], especially the chosen-plaintext attack method [31], while combing the proposed improved PSO algorithm, the rotation angles used in iterative gyrator transforms may be obtained. This will be our future work.

## 5. Conclusions

For gyrator transform-based image encryption, the rotation angle used in the gyrator transform is one of the secret keys. In this paper, considering the properties of the gyrator transform, an improved PSO algorithm was proposed and applied to search the rotation angle in a single gyrator transform. The experimental results demonstrate that the proposed improved PSO algorithm can significantly improve the efficiency of searching the rotation angle of a single gyrator transform. Since gyrator transform is the foundation of image encryption in gyrator transform domains, the research on the method of searching the rotation angle in a single gyrator transform is useful for further study on the security of the image encryption algorithms based on the gyrator transform.

The basic idea of the proposed improved PSO algorithm can also be used to search the optimization solutions for other problems with the similar characteristics of severe local convergence and obvious global fluctuations.
